# Canthotomy For the Relief of Blepharospasm

**Published:** 1879-07

**Authors:** Chas. W. Miles

**Affiliations:** Jordan, Ky.


ATLANTA
Medical and Surgical Journal.

Vol. XVII.] JULY—1879. [No. 4

### Original Communications.

### CANTHOTOMY FOR THE RELIEF OF BLEPHAROSPASM.

By CHAS. W. MILES, M.D., Jordan, Ky.

The management of blepharospasm has not received the consideration from our standard authors which is due it. To become fully impressed with the truth of this statement one needs but to refer to our best works on diseases of the eye when sorely in need of a reliable remedy for the relief of an obstinate case of this kind.

A search through the literature of the day yields about the following as the sum total in the way of treatment:—Cold water, tonics and hypodermic morphia.

After a failure of these remedies, which most usually occurs, we are taught, as a denier resort, to divide the supra-orbital nerve. This last proceedure is, however, if properly performed, sometimes followed by unfortunate results. Sea-bathing has been recommended in certain cases, but with a majority of patients living in the interior this is impracticable.

Pressure at a given point, while sometimes attended with temporary relief, rarely accomplishes permanent good.

Quite recently I have adopted the method of treating this disagreeable affection, when complicating inflammatory diseases of the eye, by canthotomy.

I use the term "canthotomy" in contra-distinction to the term canthoplasty, which latter term should be confined to the operation of cutting the outer canthus and inserting a flap of skin from the adjacent parts, a proceedure which is highly gratifying in certain cases of extreme tension of the lids. My first experience with this method of cure was in the case of a lady who had suffered with keratitis for two years, accompanied by a violent and continued blepharospasm. The lashes of the upper lid were kept constantly in contact with the globe of the eye, thus acting as an additional source of irritation. A trial of several weeks with the usual remedies utterly failing, I was induced by reading an article on the subject to try canthotomy, which I did by means of stout scissors—cutting the orbicularis and freely dissecting the underlying connective tissue from its bony attachments. The skin and conjunctiva were brought together by means of sutures and cold water dressings applied.

Immediate and entire relief of the symptoms followed, the lashes resting quietly in their natural position, and the lid easily everted by simply lifting its ciliary margin. I prefer this method of treating blepharospasm in all cases where the cause lies in some temporary disease of the eye and its appendages, especially if the disease be of an inflammatory character. Of course I would exhaust the usual means of cure before resorting to the use of the knife. I certainly would not temporize in diseases seriously affecting the globe of the eye itself, especially when we consider the safety and certainty of relief by canthotomy.

The operation possesses other and very important advantages as a direct measure of cure in cases of keratitis, granular lids, etc. Where, however, the case clearly belongs to the class of neuroses, I prefer the old method of dividing the supra-orbital nerve, which should never be limited to a simple division of the nerve, but rather a section of the nerve should be removed entirely.

In performing the operation of canthotomy, I consider it of great importance to freely sever all connection between the canthus and the edge of the orbit, as suggested by Dr. Noyes. Anyone can readily satisfy himself of the importance of this by seizing the skin in this region and attempt to lift it from the underlying bone.

The method of simply slitting the outer canthus and bringing the skin and conjunctiva together should become obsolete. It certainly does not answer for the relief of blepharospasm.

